# Circ2Traits: a comprehensive database for circular RNA potentially associated with disease and traits

**DOI:** 10.3389/fgene.2013.00283

**Published:** 2013-12-10

**Authors:** Suman Ghosal, Shaoli Das, Rituparno Sen, Piyali Basak, Jayprokas Chakrabarti

**Affiliations:** ^1^Computational Biology Group, Theory Department, Indian Association for the Cultivation of ScienceKolkata, India; ^2^GyanxetKolkata, India; ^3^School of Bioscience and Engineering, Jadavpur UniversityKolkata, India

**Keywords:** circular RNA, miRNA, interaction network, disease, SNP

## Abstract

Circular RNAs are new players in regulation of post transcriptional gene expression. Animal genomes express many circular RNAs from diverse genomic locations. A recent study has validated a fairly large number of circular RNAs in human, mouse, and nematode. Circular RNAs play a crucial role in fine tuning the level of miRNA mediated regulation of gene expression by sequestering the miRNAs. Their interaction with disease associated miRNAs indicates that circular RNAs are important for disease regulation. In this paper we studied the potential association of circular RNAs (circRNA) with human diseases in two different ways. Firstly, the interactions of circRNAs with disease associated miRNAs were identified, following which the likelihood of a circRNA being associated with a disease was calculated. For the miRNAs associated with individual diseases, we constructed a network of predicted interactions between the miRNAs and protein coding, long non-coding and circular RNA genes. We carried out gene ontology (GO) enrichment analysis on the set of protein coding genes in the miRNA- circRNA interactome of individual diseases to check the enrichment of genes associated with particular biological processes. Secondly, disease associated SNPs were mapped on circRNA loci, and Argonaute (Ago) interaction sites on circular RNAs were identified. We compiled a database of disease-circRNA association in Circ2Traits (http://gyanxet-beta.com/circdb/), the first comprehensive knowledgebase of potential association of circular RNAs with diseases in human.

## Introduction

Circular RNAs, formed by covalent linkage of the ends of a single RNA molecule, are newly discovered RNAs that sponge miRNAs to block their function (Memczak et al., 2013). These circular transcripts were previously thought to be scarcely expressed until recently, when thousands of circular RNA transcripts were identified in both human and mouse by two independent studies by Jeck et al. (2013) and Memczak et al. (2013). Like the previously reported circular RNAs in archea (Grabowski et al., 1981), unicellular organisms (Tetrahymena thermophila) (Danan et al., 2012) and plants (Sanger et al., 1976), animal genomes abundantly express many circular RNAs from diverse genomic locations, such as coding and non-coding exons, intergenic regions or transcripts antisense to 5′ and 3′UTRs (Jeck et al., 2013). In the study by Jeck et al. (2013), circular RNAs were identified by high throughput sequencing of the ribosome-depleted fraction of RNA treated by RNase R (RNase R exonuclease degrades linear RNAs but leaves circRNAs unaffected), combined with a bioinformatic algorithm. Memczak et al have identified 2000 human, 1900 mouse, and 700 nematode circular RNAs from sequencing data (Memczak et al., 2013). A number of the identified circular RNAs were further experimentally tested by Memczak et al in HEK293 cells. Often the expressions of these circular RNAs show tissue specificity or even specificity to developmental stages. A study by Capel et al found circular RNA in Sex Determining Region Y (SRY) which is highly expressed in testes (Capel et al., 1993). Circular non-polyadenylated RNA molecules have been identified as stable transcription products of the human ETS-1 and mouse Sry genes (Dubin et al., 1995). Jeck et al suggested that endogenous circular RNAs are abundant, stable, conserved and non-random products of RNA splicing and may have roles to play in control of gene expression. Using high-throughput sequencing libraries they have identified more than 25,000 distinct RNA species in human fibroblasts that contain non-collinear exons and were reproducibly enriched by exonuclease degradation of linear RNA (Jeck et al., 2013).

The fact that circular RNAs are targeted by endogenous miRNAs was reported by Hansen et al. The authors outlined a circRNA destruction mechanism in which miR-671 binds CDR1as/ciRS-7 with greater complementarities than miR-7 and induces cleavage of this circRNA mediated by Ago [the catalytic component of RNA induced silencing complex (RISC) which is a central component of RNA interference (RNAi) machinery] (Hansen et al., 2011). Furthermore, it has recently come to light that these circular RNAs play a critical role in fine-tuning the level of miRNA mediated regulation of gene expression by sequestering the miRNAs. A study has identified a highly expressed endogenous circular RNA (circRNA) in human brain and mouse brain and has demonstrated that the circRNA in Cerebellar Degeneration Related protein 1 (CDR1) locus can be endonucleolytically cleaved by miR-671 in an Ago2- dependent manner (Hansen et al., 2011, 2013a,b). This phenomenon leads to decrease in CDR1 mRNA levels independently of heterochromatin formation.

Owing to their ability for sequestration of miRNAs, circular RNAs play an important role in fine tuning of post-transcriptional gene expression. From their interaction with disease associated miRNAs, we surmise they have a potential role in disease regulation. In the study by Hansen et al. (2011), the circRNA CDR1as, which is the antisense transcript of CDR1, has been identified to be targeted by miR-7, a miRNA implicated in various disease, including Parkinson's disease and several types of cancers. Importantly, the circRNA CDR1as was seen to be frequently expressed in many tumor cell lines including neuroblastoma (widespread expression), renal cell, and lung carcinomas (Hansen et al., 2013a,b). Its expression was primarily found in the brain where it was co-expressed with miR-7 (Hansen et al., 2011). In the current paper we studied the potential association of circular RNAs with human diseases by two different approaches. Firstly, we studied the potential interactions of circular RNAs with disease associated miRNAs and constructed the interactome network (comprising of protein coding, non-coding, and circular RNA genes) of miRNAs associated with individual diseases. We implemented a measure for the likelihood of a circRNA to be associated with a disease in terms of the statistical significance of the circRNA's interaction with miRNAs associated with the concerned disease. Gene ontology (GO) enrichment analysis was performed on the set of protein coding genes in the miRNA-circRNA interactome of diseases to check the enrichment of genes associated with particular biological processes. Secondly, we focussed on disease and traits associated variations in the circular RNA loci and also mapped Ago interaction sites within circular RNA loci. We compiled a database Circ2Traits, the first comprehensive knowledgebase for potential association of circular RNAs with diseases in human.

## Materials and methods

### Data source

We used the circular RNA dataset from Memczak et al. (2013). This dataset consists of 1953 predicted human circular RNAs along with their genomic coordinates, annotation, and predicted miRNA seed matches. Disease related miRNA data was taken from miR2disease (Jiang et al., 2009) to check the association of circular RNAs with disease associated miRNAs. miR2Disease currently stores curated information about human miRNAs associated with 174 different diseases in human. From Starbase (Yang et al., 2011) we collected the miRNA-mRNA interaction data predicted by MiRanda (Enright et al., 2003), TargetScan (Grimson et al., 2007), PiTA (Kertesz et al., 2007), PicTar (Krek et al., 2005), and RNA22 (Miranda et al., 2006) that mapped to the Ago interacting regions. Dataset for predicted miRNA and long non-coding RNA interaction pairs was collected from miRCode database (Jeggari et al., 2012).

### Calculating the likehood of circRNAs to be associated with diseases

We implemented a measure for the likelihood of a circRNA to be associated with a disease in terms of the significance of the circRNA's interaction with miRNAs associated with the concerned disease. We calculated *p*-values for disease association of each circRNAs interacting with disease associated miRNAs. We conducted hypergeometric test using the following parameters:

- number of disease associated miRNAs interacting with the circRNA against the total number of disease associated miRNAs
- the total number of miRNAs interacting with the circRNA
- the total number of human miRNAs

For each circRNA interacting with miRNAs associated with a particular disease, the *p*-value for the likelihood of the circRNA to be enriched for interaction with miRNAs associated with that disease is calculated as:

(1) $$p=\sum_{i\;=\;m_{d}}^{\min}\left(m_{c},M_{d}\right)\frac{\left(\begin{array}{l} M_{d}\\ i \end{array}\right)\left(\begin{array}{l} M_{T}-M_{d}\\ m_{c}-i \end{array}\right)}{\left(\begin{array}{l} M_{T}\\ m_{c} \end{array}\right)}$$

Where,

*M_T_* = Total number of miRNAs in the human genome

*m_c_* = Number of miRNAs interacting with the circRNA

*M_d_* = Total number of miRNAs associated with diseased

*m_d_* = Number of miRNAs associated with disease d that interact with the circRNA.

After applying Bonferroni correction, a circRNA with a *p*-value < *p*_threshold_ is shown as likely to be associated with the concerned disease, where,

(2) $$p_{\text{threshold}}\text{=0}.\text{05/}m$$

*m* = Total number of circRNAs interacting with any miRNA associated with the concerned disease.

### Construction of miRNA interaction networks

We used the predicted mRNA, lncRNA, and circular RNA targets of all microRNAs associated with individual diseases to construct the networks depicting the regulation of post-transcriptional gene expression for each of the diseases. The networks were constructed using cytoscape (Shannon et al., 2003) by giving as input the miRNA-target interaction table in the standard two column format consisting of miRNA-circRNA, miRNA-mRNA, and miRNA-lncRNA interaction. We performed GO enrichment analysis on the set of protein coding genes in the miRNA and circRNA interactome networks of individual diseases using GORILLA GO enrichment analysis tool (Eden et al., 2009).

### Identification of GWAS associated SNPs and ago interaction sites within circrnas

We used Ago interaction sites from Ago PAR-CLIP data from the dataset of Hafner et al. (2010), for mapping Ago interaction sites within circular RNA loci. For studying variations within the circular RNA loci, we collected genome wide SNP data from dbSNP at NCBI (Sherry et al., 2001). Traits associated SNP dataset was collected from the GWAS catalog of NHGRI that has a total of 5807 variations associated with 540 traits (Hindorff et al., 2009). We executed all the mappings through custom programs developed in JAVA. The web interface and database were designed in PHP and MySQL.

## Results

### Association of circular RNAs with disease associated miRNAs

We analyzed human disease associated miRNA data collected for 174 diseases to find circular RNAs interacting with these disease-associated miRNAs. For 105 diseases, including several carcinomas and neurodegenerative diseases, we found such connections with circular RNAs (see supplementary file 1). We implemented a measure for showing the likelihood of a circRNA to be associated with a disease in terms of the significance of the circRNA's interaction with miRNAs associated with the concerned disease. While searching for the potential disease associated circRNAs, the users will see only the circRNAs with greater likelihood (measured by *p*-value calculated using hypergeometric test; see the Materials and Methods section) for the disease association (in the form of their enrichment for interacting with miRNAs associated with the concerned disease).

**Figure 1:**
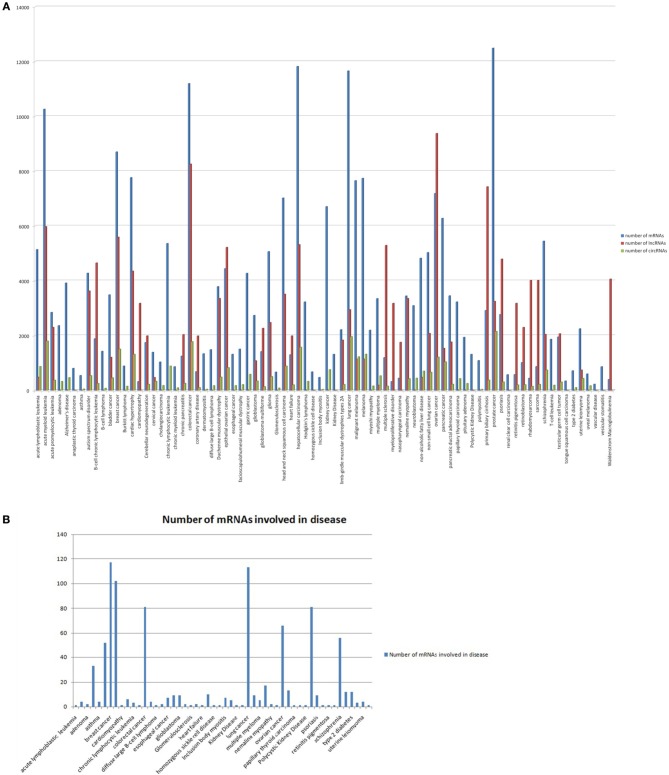
**(A)** The number of mRNAs (blue), lncRNAs (red), and circRNAs (green) interacting with miRNAs associated with individual diseases. The number of mRNAs, lncRNAs, or circRNAs is plotted along the Y-axis, while the X-axis shows the individual disease names. **(B)** The number of genes interacting with disease associated miRNAs (as identified in our study) that overlapped with the disease associated genes in Genetic Association database for individual disease.

To get a comprehensive picture of post transcriptional regulation in these diseases, we collected the mRNA and lncRNA targets of the disease associated miRNAs. The mRNA targets of human miRNAs predicted from PAR-CLIP data (Hafner et al., 2010) by widely used miRNA prediction tools, such as TargetScan, miRanda, PicTar, PITA, and RNA22 were collected from StarBase database. This dataset is more reliable, as the predictions are limited within Ago interacting regions. Alongside mRNA targets, miRNAs are shown to target long non-coding RNAs (lncRNAs) and pseudogenes. These non-protein coding targets of miRNAs also play a critical role in sequestration of miRNAs and thus limit the level of the available cellular miRNAs for targeting other protein coding targets (Wang et al., 2010; Cesana et al., 2011; Tay et al., 2011). Hence, for modeling the miRNA mediated post transcriptional regulatory network in cells, it is important to add the lncRNA and circRNA components along with the mRNAs. We constructed networks for miRNA mediated post transcriptional repression in 105 diseases those were found to be associated with circRNAs, i.e., any of the disease associated miRNAs that was found to have binding sites in circRNAs. The number of circRNAs, protein coding RNAs and lncRNAs interacting with miRNAs associated with individual diseases is plotted in Figure 1A (circRNAs in green, protein coding RNAs in blue and lncRNAs in red).

Furthermore, we searched for the protein coding genes for individual diseases that were already reported to be associated with the diseases by different functional studies. The data for disease associated genes were collected from the Genetic Association database (Zhang et al., 2010). The Genetic Association database mines disease association information collected from published papers and GWAS studies. Figure 1B shows the number of disease associated genes in our study that overlapped with the disease associated genes in the Genetic Association database for individual diseases.

**Figure 2:**
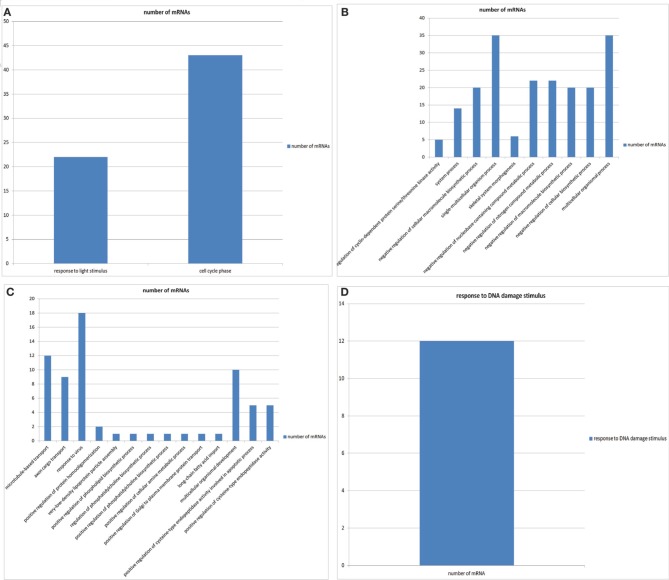
**(A)** The GO enrichments of genes associated with breast cancer. **(B)** The GO enrichments of genes associated with cervical cancer. **(C)** The GO enrichments of genes associated with gastric cancer. **(D)** The GO enrichments of genes associated with oral cancer.

We performed GO enrichment analysis for mRNA interactome of circRNAs for individual diseases to check if the genes associated with circRNAs are preferentially associated with particular biological processes. We found such enrichment for biological processes for mRNAs in 90 diseases (see supplementary file 1). Figures 2A–D show the GO enrichments of genes associated with breast cancer, cervical cancer, gastric cancer and oral carcinoma, respectively.

To enhance the reliability for disease association, we provided a filtering step where users can view only the interaction networks of miRNAs that have a defined and validated role in a particular disease, i.e., not all the miRNAs with just differential expression signatures. For instance, miR-193b is characterized to have a defined role in breast cancer as its down-regulation contributes to tumor progression and invasion (Li et al., 2009). We have implemented this feature by manual curation of disease associated miRNAs.

### Association of circular RNAs with traits associated SNPs

**Figure 3:**
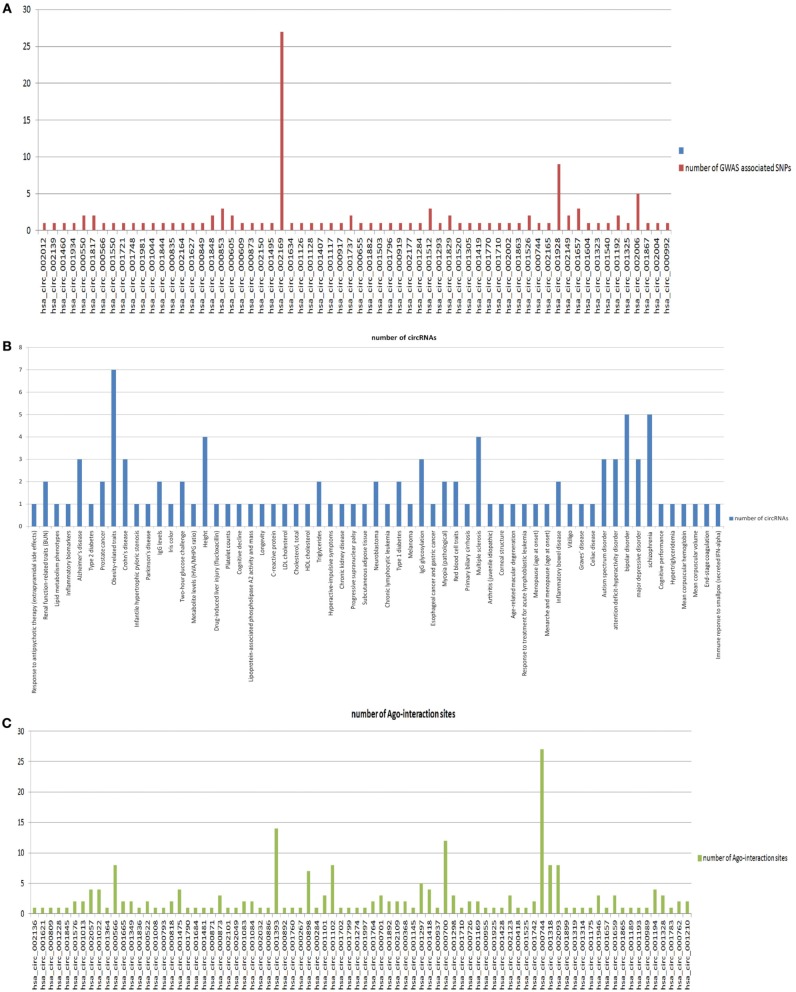
**(A)** The number of traits associated SNPs mapped into each of the 64 circRNAs. **(B)** The distribution of circRNAs associated with these 61 different traits. **(C)** The distribution of Ago interacting sites on 82 circRNAs.

For further understanding of circRNA functions and their disease or traits association, we looked for variations in the circRNA loci. We mapped the loci of variations in human genomic sequence (collected from dbSNP) into the genomic loci of circRNAs. The average number of variations mapped into 1951 circRNA loci was 217.13 and the frequency of variations per kilobase (kb) was 24.01. This result indicates that circRNAs are much less conserved than the protein coding RNAs, as the frequency of variations in circRNAs is much higher than that of the CDS (0.37 SNP per kb) and UTR (0.46 SNP per kb) of protein coding RNAs (Bhartiya et al., 2013). We further inspected SNPs associated with diseases and traits in the circRNA loci. We found 93 unique GWAS associated SNPs mapped into 64 out of 1951 circRNAs (data in supplementary file 2). Average number of GWAS associated SNPs mapped per circRNA for those 64 circRNAs was 1.828. Figure 3A shows the number of traits associated SNPs mapped into each of the 64 circRNAs. The GWAS associated SNPs in circRNAs include SNPs associated with 61 different traits including several types of cancers and neurodegenerative diseases. Figure 3B shows the distribution of circRNAs associated with these 61 different traits.

### Ago interaction sites in the circRNA loci

As circRNAs are found to predominantly function as miRNA sponges, it is evident that their functional sites are the sites of miRNA binding, i.e., Ago binding sites. Firstly, we mapped the Ago binding sites from the CLIP-SEQ dataset of Hafner et al to the circRNA loci. Among 1951 circRNAs, we found 82 circRNAs whose genomic loci overlapped with one or more Ago-interacting regions from Hafner dataset (data in supplementary file 3). The average number of Ago-binding sites mapped per circRNA was 2.658. Next, we sought to find variations in the Ago-binding sites within circRNAs. We found that among 82 circRNAs with Ago-binding sites, 49 circRNAs contain one or more variations in their Ago-binding sites (data in supplementary file 4). The average frequency of SNPs in Ago binding sites on 49 circRNAs is 3.1224. Figure 3C shows the distribution of Ago interacting sites on 82 circRNAs. We did not find any GWAS associated SNPs mapped into the Ago-binding regions within circRNAs.

### Usage of circ2Traits

**Figure 4:**
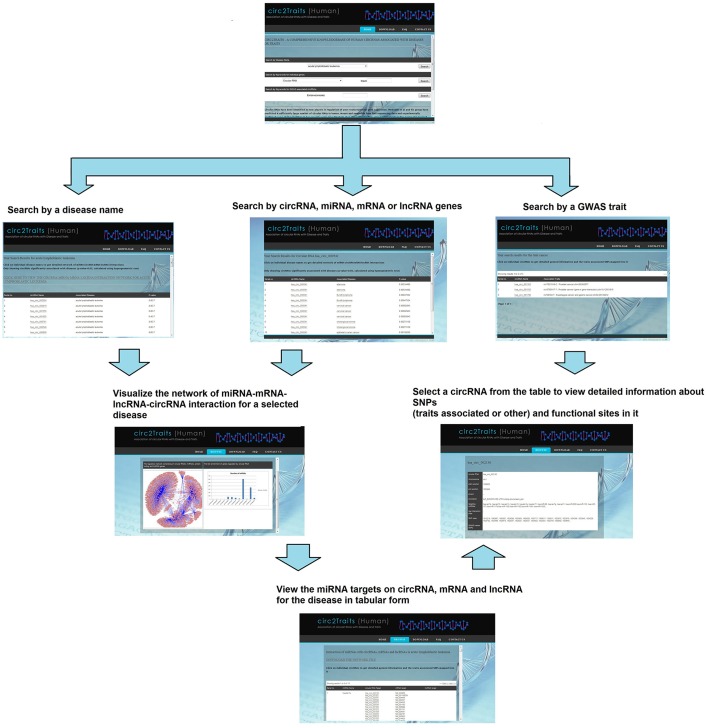
**A flow diagram showing the usage of circ2Traits database**. In the first step, users can search by either disease name, or circRNA/miRNA/mRNA/lncRNA identifier, or traits by keyword. The results page will show the circRNAs/miRNAs/mRNAs/lncRNAs potentially associated with disease or traits. In the second step, further searching by particular disease name will show the user the whole miRNA-target interaction network for the particular disease. Searching by particular circRNA will show the detailed information about the circRNA.

Circ2Traits (http://gyanxet-beta.com/circdb/) stores information about circRNAs, categorized according to their potential association with diseases, as observed from the GWAS associated SNPs and potential interaction with disease associated miRNAs. The present version of circ2Traits has categorized 1951 human circRNAs potentially associated with 105 different diseases. Furthermore, circ2Traits stores the complete putative miRNA-circRNA-mRNA-lncRNA interaction network for each of these diseases. To begin with, the users can choose from a directory of the 105 diseases, to view a list of circRNAs most likely to be associated with the disease and also visualize the interaction network and see the interaction table for each disease. There are other search options like keyword based search for miRNAs, circRNAs, protein coding genes (symbols or mRNA accession), and lncRNAs. Search options for GWAS traits associated circRNAs are also available. For each circRNA, information like traits associated and other SNPs, Ago interaction sites are stored besides general information about the circRNA (name, locus, and interacting miRNAs). The information on individual circRNAs can be viewed upon selecting a circRNA from the interaction table. Figure 4 shows the usage of circ2Traits pictorially.

## Discussion

circRNAs, stable transcripts expressed from diverse genomic locations, are recently identified as important players in regulation of cellular miRNA abundance and thus are a major component in the miRNA-mediated post transcriptional regulatory network. These transcripts form a hidden layer in the miRNA-mediated gene expression regulation circuitry which needs to be uncovered in order to understand the post-transcriptional regulations in cells. Available reports suggest the circRNAs show tissue specific and developmental stage specific expressions. Their interactions with miRNAs imply that possibly they are associated with many diseases. Recent availability of a considerable number of high confidence human circRNA transcripts from RNA Sequencing prompted us to carry out our work for categorization of these circRNAs according to their potential involvement with diseases. We searched for potential disease associated circRNAs by two different approaches; firstly through the predicted interaction of circRNAs with disease associated miRNAs and secondly through the association of circRNAs with disease related SNPs. We implemented a measure for showing the likelihood of a circRNA to be associated with a disease in terms of the significance of the circRNA's interaction with miRNAs associated with the concerned disease. While searching for the potential disease associated circRNAs, the users will see only the circRNAs with greater likelihood (see the Materials and Methods section) for possible disease association in the form of their enrichment for interacting with miRNAs associated with the concerned disease. Our study revealed association of circRNAs with SNPs related to a wide range of diseases including gastric, oesophageal and prostate cancers and neurodegenerative diseases like Parkinson's disease, Alzheimer's disease, Multiple sclerosis, schizophrenia among many others. Further, we checked for variations in the functional sites within circRNAs, in the Ago-interaction sites within them. We mapped Ago-interaction sites from CLIP-SEQ datasets into circRNA genomic loci and searched for variations within these sites. We developed the post transcriptional regulatory network for 105 human diseases comprising miRNAs, circRNAs, mRNAs, and lncRNAs. All these information are compiled into the database circ2Traits, the first comprehensive knowledgebase for potential disease association of circRNAs.

The study by Hansen et al. (2011) revealed overlaps in expression of the circRNA CDR1as and miR-7 in mouse brain suggesting that a major amount of miR-7 is attached to CDR1as in mouse brain. Interestingly, our database identifies the circRNA CDR1as most likely to be associated with the neurological disorder Parkinson's disease. Parkinson's disease results from the degeneration of dopamine-generating cells in midbrain and disruption of miRNA network in these cells supposedly play a critical role in the disease pathogenesis. In neurons, miR-7 was identified as a regulator of alpha-synuclein, whose over-expression is associated with impaired function of dopamine-generating cells and development of Parkinson's disease. Loss of miR-7 in cultured Parkinson's disease cells possibly contributes to increased alpha-synuclein level (Junn et al., 2009). Interestingly, in accordance with our predicted association of CDR1as with Parkinson's disease, CDR1as is mostly expressed in brain. This illustration from our database could be important starting point for researchers for further investigations in this direction.

In the future versions of the database we will incorporate more elaborate study of the binding sites for the different RNA-binding proteins in the circRNA loci. As the field matures, and the expression levels of circRNAs become more precisely known, we hope to include the tissue specific expression patterns of the circRNAs (whenever available) in our database to make it a more comprehensive and useful tool.

## Author contributions

Suman Ghosal conceived the idea of the work. Suman Ghosal, Shaoli Das, and Rituparno Sen performed the analysis of data. Rituparno Sen and Shaoli Das implemented the web interface. Suman Ghosal, Shaoli Das, and Jayprokas Chakrabarti wrote the manuscript. Piyali Basak co-supervised the data analysis. Jayprokas Chakrabarti supervised the whole work.

### Conflict of interest statement

The authors declare that the research was conducted in the absence of any commercial or financial relationships that could be construed as a potential conflict of interest.
